# Climate‐Action Driven Emission Changes of Unintentional Persistent Organic Pollutants From Global Copper Industry

**DOI:** 10.1002/gch2.70153

**Published:** 2026-08-21

**Authors:** Yuxiang Sun, Qiuting Yang, Jianghui Yun, Yujue Yang, Haiying Yu, Minghui Zheng, Guorui Liu

**Affiliations:** ^1^ Zhejiang Key Laboratory of Digital Intelligence Monitoring and Restoration of Watershed Environment College of Geography and Environmental Sciences Zhejiang Normal University Jinhua China; ^2^ State Key Laboratory of Environmental Chemistry and Ecotoxicology Research Center for Eco‐Environmental Sciences Chinese Academy of Sciences Beijing China; ^3^ College of Resource and Environment University of Chinese Academy of Sciences Beijing China; ^4^ School of Environment Hangzhou Institute for Advanced Study, UCAS Hangzhou China; ^5^ Hubei Key Laboratory of Environmental and Health Effects of Persistent Toxic Substances School of Environment and Health Jianghan University Wuhan China

**Keywords:** climate change, corporate governance, decarbonylation, east asia, environmental protection, environmental science, natural resource economics, pollutant, smelting

## Abstract

Climate action is shifting copper production from primary copper smelting (PCU) toward secondary copper smelting (SCU_RF), an important source of unintentional persistent organic pollutants (UPOPs). However, climate‐driven changes in UPOP emissions across the copper industry remain poorly understood. Here, emissions of six Stockholm Convention‐listed UPOPs from global and regional copper‐smelting subsectors during 2023–2050 are estimated under different climate scenarios. Toxic equivalent (TEQ)‐based emissions are used to compare relative toxic potency, while mass emissions are also quantified to reflect non‐TEQ‐based management relevance. By 2050, global UPOP emissions under a net‐zero pathway are projected to be 10%–31% higher than under the current‐policy pathway. The TEQ emissions of polychlorinated dibenzo‐p‐dioxins and dibenzofurans (PCDD/Fs) and polychlorinated biphenyls (PCBs), together with mass emissions of hexachlorobenzene (HCB) and pentachlorobenzene (PeCBz), warrant attention. SCU_RF is projected to contribute 62%–98% of total TEQ emissions across UPOPs, making it the priority control source, while mass emissions of HCB and PeCBz from PCU and direct melting of high‐quality scrap should receive attention. Climate‐action‐driven TEQ emission increases are concentrated in East Asia and the Pacific and in Europe and Central Asia. These findings reveal a pollution trade‐off in copper decarbonization and highlight the need for targeted control strategies.

## Graphical abstract

Climate action profoundly reshapes global copper production by increasing output and altering the production technology mix. These changes also raise emissions of unintentional persistent organic pollutants (UPOPs) from the copper industry, revealing a hidden pollution trade‐off that should be addressed alongside decarbonization.

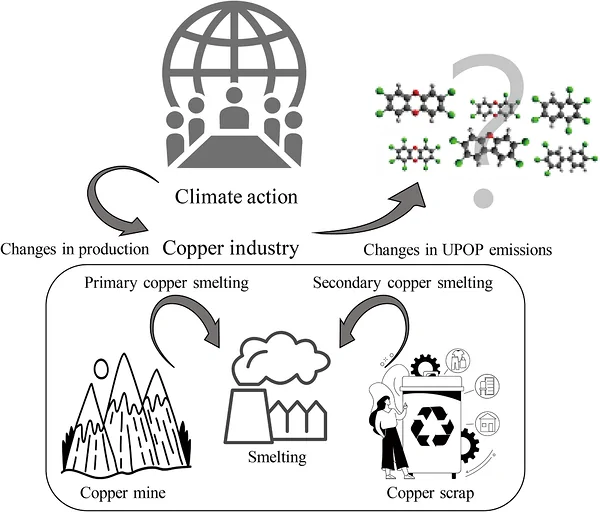

## 1 Introduction

Copper is a critical material intensively used in decarbonization technologies. Large amounts of copper are needed for technological solutions expected to deliver two‐thirds of greenhouse gas emission reductions [1]. For instance, in low‐carbon energy systems, even excluding electricity storage and transmission requirements, a 1 megawatt solar power system and a 3 megawatt wind turbine require 5.5 and 4.7 tons of copper [2], respectively. At the same time, the copper sector faces growing pressure to decarbonize while ensuring copper supply security, accelerating a shift from primary production to recycling‐based supply [3]. Through secondary copper smelting process (SCU_RF), greenhouse gas emissions can be reduced by up to 85% compared to primary copper smelting process (PCU) [4]. According to the International Copper Association, global refined copper production will double by 2050, with an increasing share from secondary smelting processes [1] with the aim of achieving net‐zero emissions in both the copper industry and the wider economy. The copper industry is one of the most indispensable and climate‐sensitive metal sectors for achieving climate targets.

However, beyond carbon, the copper industry has raised long‐standing concerns about unintentionally produced persistent organic pollutants (UPOPs) [5, 6, 7, 8, 9]. Polychlorinated dibenzo‐*p*‐dioxins and dibenzofurans (PCDD/Fs), polychlorinated biphenyls (PCBs), polychlorinated naphthalenes (PCNs), hexachlorobeNEZne (HCB), pentachlorobeNEZne (PeCBz), and hexachlorobutadiene (HCBD) are typical UPOPs covered by Stockholm Convention [10]. Those UPOPs have been associated with human health risks of breast, prostate and colorectal cancers [11, 12, 13, 14]. UPOPs can be generated during copper smelting through incomplete combustion of organic residue including cables [15, 16, 17] and reformation during the cooling stage of flue gas [18, 19, 20]. Previous studies have proven that copper is one of the most effective catalytic elements promoting the formation of UPOPs [18, 20, 21]. The emission intensity of PCDD/Fs and PCBs from SCU_RF is higher than that of steelmaking, aluminum smelting, and other non‐ferrous metal smelting processes [22]. Although studies quantifying the global contribution of copper smelting remain limited, metal production is recognized as an important source of UPOPs. Taking PCDD/Fs as an example, the metal industry accounts for more than 30% of global emissions [23]. Regional studies further indicate that copper smelting can make a substantial contribution to UPOP emissions within the metal‐smelting sector. Secondary copper smelting has been identified as one of the six major PCDD/F emission source categories in North America [24]. In China, secondary copper production is the third‐largest source of PCDD/F emissions, contributing 8% of national emissions, a share comparable to that of municipal solid waste incineration (8.6%) [25], and accounting for 70.45% of PCDD/F emissions from the secondary non‐ferrous metal industry [26]. In Korea, copper production contributes more than 90% of PCDD/F and PCB emissions from metallurgical activities [27]. Therefore, copper smelting is one of the priority industrial sectors for UPOP monitoring and control [13, 22, 28].

Inventory of UPOP emissions from multiple countries or regions from SCU_RF have been reported [15, 29, 30]. However, existing studies remain insufficient to support an assessment of climate‐action‐driven changes in UPOP emissions from the copper industry. (i) Changes in copper production under climate scenarios still need to be systematically integrated. Many climate‐policy and mitigation‐strategy studies have examined changes in copper demand, production levels, and supply patterns required to meet future climate targets. However, these models differ substantially in scenario architecture, statistical scope, subsector coverage, modelling approach, and transparency [3, 31, 32, 33, 34, 35], making it difficult to directly translate their results into activity data for UPOP emission assessment. (ii) The sectoral boundaries of existing UPOP emission inventories are not yet fully aligned with the process categories used in climate‐related copper production pathways. Their coverage does not extend to other smelting processes in the copper industry, including PCU and direct melting process of high‐quality copper scrap (SCU_DM) [5, 8, 16, 22]. Although these processes could remain important sources of copper supply, the UPOP emission factors (EFs) for these processes are still poorly constrained. (iii) Existing studies have primarily documented the current emission status [9, 30, 36, 37] without considering climate‐action‐driven changes in copper production, which could alter the sub‐sectoral distribution, process‐level contributions, and control priorities of UPOP emissions from copper smelting. As a result, how climate‐action‐driven transitions in the copper industry reshape its UPOP emissions remains unclear [38]. Clarifying these changes is essential for bridging the existing climate [39] and UPOP [10] policies as well as the development of coordinated pollution control strategies.

In this study, we synthesized global copper‐development trends and compile scenario‐consistent projections of copper output and structural shifts for 2023–2050 under different climate scenarios (Texts S1 and S2). EFs for six regulated UPOPs were derived across all copper‐smelting subsectors based on a structured literature review and statistical analysis (Text S3). The changing trends of global UPOP emissions from the copper industry under different levels of climate‐policy ambition were assessed, and the key emitting subsectors and regional hotspots were identified. These results could be helpful for achieving coordinated UPOP control as the copper industry transitions toward climate‐aligned development.

## 2 Materials and Methods

### 2.1 Copper Production Under Different Climate Scenarios

Global copper smelting can be grouped into three subsectors, PCU, SCU_RF, and SCU_DM, defined by feedstock type [40, 41]. PCU uses copper concentrate and requires smelting and subsequent converting and refining to produce copper. SCU_DM directly remelts high quality scrap for recycling. By contrast, SCU_RF processes lower quality scrap containing organic residues, such as insulated cables and wires, and is processed through refining.

Future copper production has been well modelled by official agencies [1, 3, 35] and academic studies [31, 32, 33, 34, 42, 43, 44]. In this study, a systematic review of published scenario projections of copper production is conducted (Text S1), from which three representative scenarios were identified. The Stated Policies Scenario (STEPS) reflects the trajectory implied by today's policy settings and stated implementation plans. The Announced Pledges Scenario (APS) incorporates countries’ announced climate targets and commitments, assuming they are fully achieved on time. The Net Zero Emissions by 2050 Scenario (NEZ) represents a stringent pathway consistent with global net zero CO_2_ emissions by mid‐century. The three scenarios were selected because they directly represent different levels of climate‐policy ambition, thereby providing a policy‐interpretable framework for comparing the relationship between climate‐action intensity and UPOP emissions. For each scenario, production estimates were compiled for 2023, 2030, 2040, and 2050 (Table S1).

Regional copper production was modelled according to the World Bank regional classification, including North America (NA), East Asia and Pacific (EAP), Europe and Central Asia (ECA), Latin America and the Caribbean (LAC), Middle East and North Africa (MENA), South Asia (SA), and Sub‐Saharan Africa (SSA). Evidence suggests that the global supply pattern has remained relatively stable in recent years [45], whereas future regional production would be shaped by multiple factors, including international trade, policy and investment decisions, resource endowments and scrap availability, leading to uncertainty. Regional copper production from 2023 to 2050 was therefore discussed under an assumption of a stable global production pattern (Text S2). Detailed production data are provided in Tables S2–S4.

### 2.2 Scenario‐Based Estimation and Uncertainty of UPOPS Emissions From 2023–2050

The six UPOPs including PCDD/Fs, PCBs, HCB, PeCBz, PCNs and HCBD were assessed because they are listed as unintentionally produced POPs under Annex C of the Stockholm Convention [10], which are highly toxic and widely regulated industrial UPOP emissions by the international community. The EF method, recommended by the European Monitoring and Evaluation Programme, the European Environment Agency [28], the U.S. Environmental Protection Agency [46] and UNEP [22], is employed in emission assessment at both national [15, 30, 36] and global scales [22]. EFs for UPOPs were determined by synthesizing available official inventories and guidelines for each copper subsector. When official data were unavailable, peer‐reviewed measurements were averaged or statistically processed to derive representative EFs (Figure S1). For PCDD/Fs, PCBs and PCNs, toxic equivalent (TEQ) was used as the reporting unit because it is the official and widely adopted metric in international emission inventories and guidelines [22, 28, 47]. TEQ‐based EFs reported in field monitoring studies and official inventories for these UPOPs are used directly. For HCB, PeCBz and HCBD, EFs were first obtained on a mass basis. To enable toxicity‐based comparison across different UPOPs, we further converted the values into TEQ‐based values using inhalation toxicity equivalency factors relative to TCDD [48, 49]. Details about EF determination and emission estimation could be seen in Texts S3 and S4.

Uncertainty primarily arises from the production scenarios and EFs. On the production side, a systematic review of available scenario projections from official agencies [1, 3, 35] and academic studies [31, 32, 33, 34, 42, 43, 44] is conducted to derive global copper production ranges under the STEPS, APS and NEZ scenarios. These ranges were used to capture scenario uncertainty in future production levels and to derive higher and lower estimates. For regional production, this study assumes a stable global supply pattern (Table S5). This assumption is consistent with the relatively stable global supply structure observed in recent years [45], while avoiding highly uncertain influences from multiple factors. It therefore provides a transparent baseline for regional assessment. Nevertheless, future changes in regional production shares could affect the spatial distribution of UPOP emissions. The current regional assessment should be interpreted as an initial comparison of regional emission patterns. There is a need for more detailed region‐specific projections of copper production under climate‐action pathways to support UPOPs emission accounting. On the emissions side, the EF method is aligned with approaches used by government agencies [28, 46], international organizations [22] and prior studies [9, 37]. EFs are primarily derived from UNEP guidance [22] and the existing literature (Table S6). To quantify the EF uncertainty of UPOPs for SCU_RF, we used the well‐documented EFs of PCDD/Fs for the SCU_RF process (Text S3) to perform probability distribution fitting and Monte Carlo simulation, from which the 2.fifth‐97.fifth percentile range and relative variability were derived. Given the similar formation mechanisms and correlated emission characteristics of PCDD/Fs and other UPOPs, and considering the limited availability of species‐specific EF data for other UPOPs, the same relative lower and upper variability derived from PCDD/F EF distribution was applied to the central EFs of different UPOP species determined from the UNEP Toolkit and literature evaluation, thereby constructing the 95% uncertainty intervals for SCU_RF EFs (Table S7). For other copper‐production processes, the availability of EF data is limited. Therefore, the literature mean was used as the central estimate, while the reported minimum and maximum values were used to characterize the uncertainty range (Table S7). In addition, future improvements in pollution‐control technologies could further change UPOP emission intensities, highlighting the need for continuous field measurements to update process‐specific EFs.

To quantify the uncertainty in emission estimates, a hybrid uncertainty matrix is developed to combine scenario‐based activity uncertainty (Table S1) with probabilistic EF uncertainty (Table S7). The activity‐level ranges and EF uncertainty intervals were then combined to calculate global and regional UPOP emissions under different uncertainty combinations. The main text discusses the results based on central EFs and scenario‐based activity‐level uncertainty, whereas the detailed results for all combinations are provided in Data S1 and S2. The results show that although absolute emission levels were affected by variations in EFs and activity levels, the overall emission trends, major contributing processes and subsector‐level emission shares remained generally consistent across uncertainty cases.

## 3 Results and Discussion

### 3.1 Climate Action‐Driven Changes of Global UPOP Emissions

**FIGURE 1:**
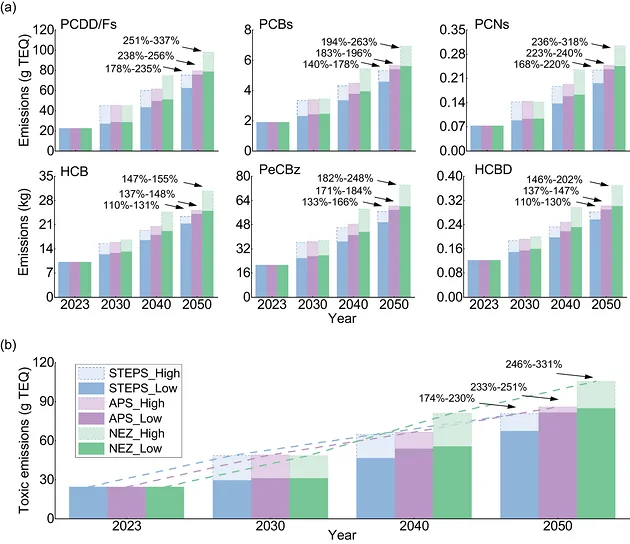
Global emissions of (a) mass of six UPOPs and (b) total toxic equivalents (TEQ) of the six UPOPs from 2023 to 2050 under STEPS, APS and NEZ (Percentages in figures indicate the relative increase in 2050 emissions compared with 2023 level).

Global atmospheric emissions of UPOPs from copper smelting were projected to rise markedly under all scenarios from 2023 to 2050, with higher emissions under more stringent climate pathways (Figure 1a). Under STEPS, emissions of the six UPOPs in 2050 were estimated at 62–75 g TEQ for PCDD/Fs, 4.58–5.31 g TEQ for PCBs, 0.20–0.23 g TEQ for PCNs, 21–23 kg for HCB, 50–87 kg for PeCBz and 0.26–0.28 kg for HCBD, equivalent to 278%–335%, 240%–278%, 268%–320%, 220%–231%, 233%–266% and 210%–230% of that in 2023, respectively. Under APS, emissions in 2050 were projected to exceed 300% of that in 2023 for PCDD/Fs and PCNs, and to exceed 230% for HCB and HCBD and 270% for PCBs and PeCBz. Under NEZ, emissions in 2050 were further 10%–30% higher than under STEPS, reaching 79–98 g TEQ for PCDD/Fs, 5.61–6.94 g TEQ for PCBs, 0.25–0.30 g TEQ for PCNs, 25–31 kg for HCB, 60–74 kg for PeCBz and 0.30–0.37 kg for HCBD. To enable toxicity‐weighted comparison across UPOPs, emissions were further adjusted using inhalation toxicity equivalency factors (TEFs) relative to TCDD [48, 49]. Summed TEQ emissions in 2050 were 6%–22% higher under APS and 26%–30% higher under NEZ than under STEPS (Figure 1b), and were 246%–331% above that in 2023 under NEZ. These results suggest that pathways aligned with net‐zero targets could obviously increase the toxic burden of UPOPs from copper smelting, highlighting a potential risk trade‐off in climate‐driven transitions.

**FIGURE 2:**
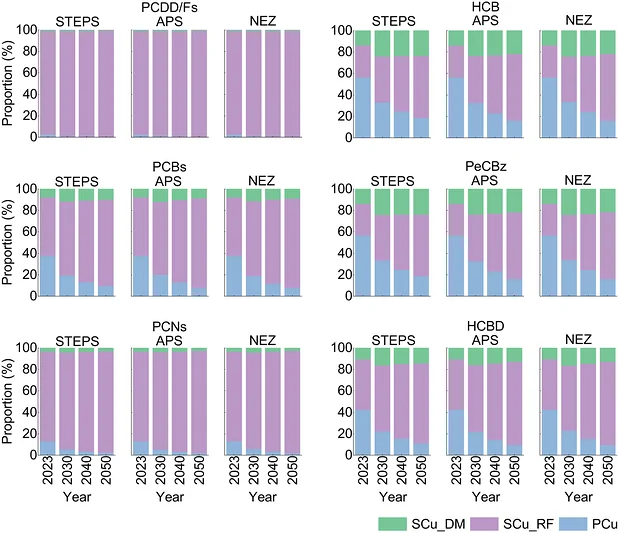
Share of global UPOP emissions from each copper smelting subsector.

From a source perspective, source‐specific emission shares (Figure 2) were calculated using the lower production estimate. The PCDD/Fs, PCBs and PCNs, were projected to be highly concentrated in SCU_RF over time (Figure 2). Across all three scenarios, SCU_RF accounted for more than 98%, 81% and 94% of global emissions of PCDD/Fs, PCBs and PCNs in 2050, respectively. By contrast, emissions of HCB, PeCBz and HCBD were highly contributed by PCU in 2023, with shares of 36%, 24% and 37%, respectively, but progressively shifted toward SCU_RF, which contributed 62%, 62% and 78% under NEZ by 2050.

### 3.2 Regional Distribution of UPOP Emissions Under Climate Scenarios

**FIGURE 3:**
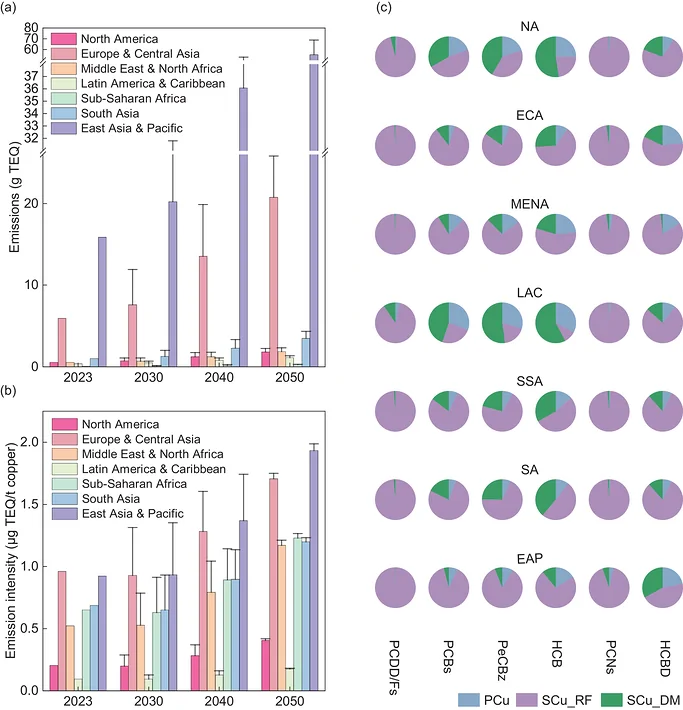
(a) Regional TEQ emissions of the six UPOPs from 2023–2050 under NEZ scenario, (b) regional TEQ emission intensity from 2023–2050 under NEZ scenario, and (c) shares of emissions from copper smelting subsectors for the six UPOPs in 2050 under NEZ scenario (NA: North America, EAP: East Asia and Pacific, ECA: Europe and Central Asia, LAC: Latin America and the Caribbean, MENA: Middle East and North Africa, SA: South Asia, and SSA: Sub‐Saharan Africa).

Full implementation of a net zero pathway was projected to drive a stronger regional emission increase than under STEPS and APS (Figure S2). For PCDD/Fs, emissions under NEZ in 2050 were projected to increase 218%‐252% of that in 2023 under the low estimate and 293%‐338% under the high estimate across regions. The NEZ pathway was projected to shift global emissions toward East Asia and the Pacific (EAP) and Europe and Central Asia (ECA), where total TEQ emissions increased by 37–50 g TEQ and 14–19 g TEQ relative to that in 2023, respectively, exceeding the increases projected for other producing regions (Figure 3a). Overall emission intensities of overall copper industry increase across all regions (Figure 3b). By 2050 under NEZ, EAP is projected to face the highest UPOP emission intensity per unit of copper production, reaching 1.93–1.99 µg TEQ/t copper, more than twice of the 2023 level. This is followed by ECA, with an emission intensity of 1.71–1.75 µg TEQ/t copper. In MENA, SA and SSA, emission intensities all exceed 1 µg TEQ/t copper by 2050, representing increases of more than 80% relative to 2023. Regional differences were also observed in the subsectoral sources of emissions (Figure 3c). In ECA, MENA, SSA, SA and EAP, SCU_RF dominated emissions of all six UPOPs. By contrast, in NA and LAC, PCU and SCU_DM were the main contributors to PCBs, PeCBz and HCB, jointly accounting for 52%‐77% in NA and 76%–90% in LAC, with SCU_DM contributing more than PCU.

### 3.3 Implications for Control of Climate Action‐Driven Emissions

Climate action could drive both rising UPOP emissions and growing regional inequality in pollution risk from copper industry. Global UPOP emissions are projected to rise under all three scenarios and to become increasingly concentrated in SCU_RF. Previous studies have pointed to emerging copper supply constraints, while also showing that recycling can help relieve supply pressure [50]. In 2023, SCU_RF and SCU_DM accounted for only 7% and 10% of global copper production, respectively, but these shares rise to 24%–29% and 29% under STEPS by 2050, and to 28%–30% and 29% under NEZ. More stringent climate pathways also imply higher copper demand because copper is essential for wind power, solar photovoltaics and electricity networks [51]. Under NEZ, global copper production in 2050 is estimated to be 9%–32% higher than under STEPS, further amplifying emissions growth. In addition to TEQ‐based priorities, mass‐based emissions of PeCBz and HCB from PCU and SCU_DM also warrant attention.

For regional UPOP emissions, larger absolute TEQ emission increases are projected for EAP and ECA, consistent with the prominent role of Asia and Europe in copper recycling and clean energy deployment [52, 53, 54, 55]. This suggests that the geography of decarbonization benefits could not coincide with that of UPOP burdens. A regional inequality would therefore emerge between contributions to climate mitigation and exposure to UPOPs related risks. This issue could be more important as rising copper recycling increases dependence on waste streams. Informal e‐waste dismantling and recycling have been linked to elevated environmental exposure and adverse health outcomes [56], while major processing and import destinations remain concentrated in regions where regulatory gaps are widespread, such as East and Southeast Asia [57] and West Africa [58]. Growing reliance on secondary copper could create hidden toxic risks along climate driven supply chains, especially where dismantling and preprocessing are weakly regulated. Notably, in regions with highly concentrated PCU, such as LAC, the mass emissions of PeCBz and HCB from primary copper production deserve attention.

As for governance and technological applications, from the perspective of priority UPOPs, PCDD/Fs remain the dominant control target when TEQ is used as the evaluation metric. However, for HCB and PeCBz, their mass emissions are substantial. HCB and PeCBz should also be regarded as important potential control targets. From the perspective of priority subsectors, SCU_RF should be prioritized because it combines rapidly increasing activity levels with relatively high UPOP emission intensities, while the HCB and PeCBz emissions from PCU and SCU_DM should also be monitored. UPOP formation in copper smelting processes is closely related to contaminated scrap inputs [15, 16, 17]. Therefore, upstream scrap‐quality standards, raw‐material admission criteria and pretreatment requirements should be strengthened. For example, previous measurements showed that an SCU_RF plant with pre‐cleaning of raw materials had an EF of PCDD/Fs as low as 0.3 µg TEQ/t [59], substantially below typical values for this process. In addition, studies indicate that UPOPs are largely emitted through particle phase [7, 59]. Improving end‐of‐pipe dust removal and flue‐gas cleaning systems could further reduce emissions. These upstream and downstream measures have the potential to offset the climate‐action‐driven increase in UPOP emissions from copper industry. From a coordinated climate‐UPOP management perspective, UPOP emissions should be explicitly incorporated into future climate‐action assessments and copper‐sector transition strategies. In practice, the expansion of SCU_RF and other subsectors should be accompanied by technology admission standards, scrap‐quality criteria, raw‐material pretreatment requirements and process‐specific implementation of best available technologies, rather than being evaluated only by its carbon‐reduction potential. Such integrated management would help avoid the transfer of carbon‐reduction benefits into unintended UPOP risks, while also further reducing carbon emissions by lowering the combustion of organic residues in scrap feedstocks.

## 4 Conclusions

These findings highlight the need for targeted control at both subsectoral and regional levels. In SCU_RF, UPOPs formation is strongly influenced by organic residues in scrap [22], indicating that stricter feedstock quality control and scrap pre‐cleaning should be a priority for UPOP control. These upstream measures could also generate climate co‐benefits by reducing the combustion of organic residues in scrap feedstocks, thereby enabling simultaneous reductions in carbon emissions and UPOPs. Lower‐emission process and control configurations are needed, including improved pretreatment, high‐efficiency particulate removal and optimized flue‐gas treatment [60]. On the demand side, scenario analyses indicate that higher material efficiency and production optimization could moderate future copper demand and thereby provide an additional pathway for reducing UPOP emissions [43] from copper smelting processes. Control priorities across copper‐smelting subsectors differ by region. SCU_RF should remain the primary control focus in most producing regions, whereas PCU and SCU_DM warrant greater attention in NA and LAC. Stronger regulation of e‐waste flows and more formalized dismantling and preprocessing systems will also be needed to reduce upstream risks.

## Author Contributions

Y.S. and G.L. conceptualized the study. Y.S., Q.Y., J.Y., Y.Y., H.Y., and G.L. collected and analyzed the data. Y.S. authored the paper. G.L. and M.Z. revised the paper. G.L. acquired funding.

## Conflicts of Interest

The authors declare no conflicts of interest.

## Supporting information




**Supporting File 01**: gch270153‐sup‐0001‐S1.xlsx.

**Supporting File 02**: gch270153‐sup‐0002‐S2.xlsx.

**Supporting File 03**: gch270153‐sup‐0003‐SuppMat.docx.

## Data Availability

The data that supports the findings of this study are available in the supplementary material of this article.
