# Supplementary material for: Climate‐Action Driven Emission Changes of Unintentional Persistent Organic Pollutants From Global Copper Industry
Source: Glob Chall. 2026 Aug 21;10(8):e70153. doi: 10.1002/gch2.70153 (PMC13494850; doi:10.1002/gch2.70153)
Supplement: Supplementary file 1 [file GCH2-10-e70153-s003.docx]

Supplementary Materials for

**Climate-action driven emission changes of unintentional persistent organic pollutants from global copper industry**

Yuxiang Sun^1,2,^^3^, Qiuting Yang^1^, Jianghui Yun^1,2,3^, Yujue Yang^1,2,3^, Haiying Yu^1^, Minghui Zheng^2,4,5^, Guorui Liu^1,3,5,*^

^1^ Zhejiang Key Laboratory of Digital Intelligence Monitoring and Restoration of Watershed Environment, College of Geography and Environmental Sciences, Zhejiang Normal University, Jinhua, China*.*

^2^ State Key Laboratory of Environmental Chemistry and Ecotoxicology, Research Center for Eco-Environmental Sciences, Chinese Academy of Sciences, Beijing, China.

^3^ College of Resource and Environment, University of Chinese Academy of Sciences, Beijing, China.

^4^ School of Environment, Hangzhou Institute for Advanced Study, UCAS, Hangzhou, China.

^5^ Hubei Key Laboratory of Environmental and Health Effects of Persistent Toxic Substances, School of Environment and Health, Jianghan University, Wuhan, 430056, China

^*^ Corresponding author, E-mail: liiugr@zjnu.edu.cn (G. Liu)

**This PDF file includes:**

Text S1-4

Figures. S1 to S2

Tables S1 to S7

**Text S1 Copper production under climate scenarios**

Although future copper production has been widely projected by official agencies and academic studies, available scenario datasets differ substantially in scenario architecture, statistical scope, subsector coverage, modelling approach and transparency. As a result, not all published projections are suitable for unintentional persistent organic pollutant (UPOP) assessment in the copper smelting sector. A systematic review of available scenario studies and reports was therefore conducted to identify datasets that could support a sector-wide assessment of climate-driven changes in UPOP emissions.

First, published studies differ in scenario families. Some are framed under SSP-based socioeconomic pathways^1-3^, whereas others use explicit climate-policy pathways such as STEPS, APS and NEZ^4-6^. The latter were considered more suitable here because they directly represent different levels of climate ambition. SSP-based copper scenarios were not used as the core framework because SSPs are socioeconomic narratives rather than explicit climate-policy pathways. They require additional study-specific assumptions to derive copper demand and supply trajectories. This leads to large differences across studies in projected copper output^3^, subsector coverage and policy interpretability, limiting their suitability for subsector-resolved UPOP assessment. Second, modelling approach was used as a screening criterion. Many studies estimate future copper demand using top-down regression models linked to GDP and population^3, 7, 8^. However, previous comparisons have shown that such methods are highly sensitive to socioeconomic assumptions and may overestimate long-term demand because they assume that the historical relationship between copper demand, GDP and population remains unchanged, while insufficiently capturing stock saturation and technology-specific material dynamics^9^. By contrast, bottom-up and stock-dynamics approaches provide stronger mechanistic constraints for long-term assessments^4-6^. Third, subsector coverage was essential for UPOP assessment. Many published studies focus on mining, primary supply or aggregate refined copper demand^1^, but do not explicitly resolve recycling pathways relevant to secondary smelting and refining. Studies limited to mine output or primary copper production were therefore insufficient on their own for a full-sector UPOP inventory. Fourth, transparency and traceability were considered. Some industry reports provide useful directional insights but rely partly on member data, proprietary commercial databases or third-party modelling frameworks. For example, the ICA pathway combines member data with Fraunhofer stock-and-flow modelling and commercial datasets such as MineSpans^10^. These sources were therefore not used as the sole modelling basis. Finally, studies that achieve large copper-demand reductions through additional resource-efficiency or behavioral measures^2, 11^ were not used as core inputs, because they represent conditional mitigation cases rather than broadly policy-defined future pathways, and would therefore yield UPOP estimates specific to those interventions rather than generally representative climate-pathway outcomes.

Based on these criteria, STEPS, APS and NEZ were selected as the core scenario framework. Production estimates for 2023, 2030, 2040 and 2050 were then compiled from IEA^5^, IFC^6^ and Born et al.^4^. These sources were prioritized because they combine explicit climate-pathway interpretation with greater relevance to copper flow structure, including the distinction between direct-use scrap and secondary production, and offer stronger comparability for subsector-resolved UPOP assessment.

**Text S2 Estimation of regional copper production from PCU, SCU_RF and SCU_DM**

The current regional share of copper production in each copper smelting sub-sector is based on the recycling input rate and copper production in 2021 published by the ICSG^12^. Share of global PCU, SCU_RF and SCU_DM production contributed by copper-producing region *f* in 2021 (SCU RF_f2021_, SCU DM_f2021_, PCU_f2021_) could be seen in Table S5.

Here, assuming a stable distribution of the global copper industry across each copper producing region, yearly copper production from SCU_RF, SCU_DM and PCU of region f in year i (SCU RF_fi_, SCU DM_fi_, PCU_fi_) is calculated by equation (1-3).

${Prop. SCU RF}_{f}={SCU RF}_{f2021}/\sum_{f} {SCU RF}_{f2021}$	(Equation 4)

${Prop. SCU DM}_{f}={SCU DM}_{f2021}/\sum_{f} {SCU DM}_{f2021}$	(Equation 5)

${Prop. PCU}_{f}={PCU}_{f2021}/\sum_{f} {PCU}_{f2021}$	(Equation 6)

${SCU RF}_{fm}={SCU RF}_{worldm}*{Prop. SCU RF}_{f}$	(Equation 7)

${SCU DM}_{fm}={SCU DM}_{worldm}*{Prop. SCU DM}_{f}$	(Equation 8)

${PCU}_{fm}={PCU}_{worldm}*{Prop. PCU}_{f}$	(Equation 9)

Where the Prop. SCU RF_f_, Prop.SCU DM_f_ and Prop.PCU_f_ mean the proportions between copper productions from SCU_RF, SCU_DM and PCU in region f (2021) and global SCU_RF, SCU_DM and PCU production. SCU RF_worldm_, SCU DM_worldM_, PCU_worldm_ represent the global production of the copper smelting sub-sectors in year m under each scenario, which is shown in Table S1.

**Text S3 Determination of emission factors (EFs) of UPOPs for PCU and SCU_RF**

**Determination of EFs of UPOPs for PCU**

Currently, information on UPOPs emissions from PCU is very limited in official documents and the literature. In the UNEP toolkit published in 2013 (https://toolkit.pops.int/), EFs for PCDD/Fs, PCBs, HCB, and PeCBz from PCU were not provided^13^. A few studies have investigated UPOP emissions from this process.

For PCDD/Fs, the U.S. EPA reported an EF of 0.31 µg TEQ/t for PCU in 1987, 1995, and 2000^14^. In 2002, Poland's Ministry of Environment reported a lower EF of 0.01 µg TEQ/t for the same process in national dioxin inventory^15^. Five years later, a study on PCDD/F emissions from two PCU plants in Poland reported EFs of 0.004 µg TEQ/t and 0.005 µg TEQ/t^16^, which were slightly lower than the EFr of 0.014 µg TEQ/t reported in a 2006 study on a PCU plant in South Korea^17^. A 2012 study investigated two PCU plants in China and found significantly varying EFs of 0.039 µg TEQ/t and 0.651 µg TEQ/t^18^, respectively. These differences were attributed to variations in production rates, feedstock composition, and pollution control equipment, although the study provided no detailed discussion. The most recent EF data for PCDD/Fs from PCU were published in the EMEP/EEA Air Pollutant Emission Inventory Guidebook (2023) (https://www.eea.europa.eu/en/analysis/publications/emep-eea-guidebook-2023), recommending an emission factor of 0.01 µg TEQ/t for this source^19^. Considering the declining trend in emissions, the consistency of most reported data, and the 2023 EMEP/EEA guidebook recommendation, this study determines an EF of 0.01 µg TEQ/t for PCDD/Fs from PCU. For PCBs, HCB, PeCBz, and PCNs, data on EFs are more limited. To our knowledge, only two studies have investigated these UPOPs. One study reported a PCB EF of 0.012 µg TEQ/t from a smelter in South Korea^17^, while another measured PCB EFs of 0.002 µg TEQ/t and 0.039 µg TEQ/t from two smelters in China^18^. The latter study also reported EFs for HCB, PeCBz, and PCNs at the same facilities. Given the limited data, the average values across different facilities for PCBs, HCB, PeCBz, and PCNs were used to estimate the final EFs in this study. A recent study conducted large-scale monitoring of HCBD emissions from PUC processes in China^20^. While no HCBD emissions were detected at two plants, emission levels at four other plants ranged from 0.5 µg/t to 2.5 µg/t, showing relatively consistent values. The final EFr for HCBD was determined as the average across all monitored facilities, at 2 µg/t.

**Determination of EFs of UPOPs for SCU_RF**

A technology split method^21^ was widely applied for developing emission inventories^13, 19, 22^, including emission inventories of UPOPs such as PCDD/Fs, PCBs, HCB, and PeCBz. In this method, the EF of UPOPs for a given source resulted from the pollution control level, and the EF for a certain level (e.g., “basic technology,” “well controlled,” and “optimized for PCDD/Fs control” for SCU_RF in the toolkit^13^ and EMEP/EEA Air Pollutant Emission Inventory Guidebook 2023^19^) did not change over time. We confirmed the UPOP EFs for SCU_RF through a two-step process. First, we assessed the current global pollution control level based on the EF change trend through a literature review. Second, EFs of UPOPs for SCU_RF under the toolkit's classification were determined based on data collection from the literature and analysis. Based on the current global pollution control levels assessed in the first step, we determined the EFs of UPOPs that best represent the global SCU_RF sector.

PCDD/Fs are the most extensively studied UPOPs emitted during SCU_RF processes, and the EFs for PCDD/Fs have shown a decreasing trend over time in the SCU_RF sector. In the *National and Regional Emissions of PCDD/PCDF* report prepared by UNEP^23^, the global SCU_RF EF for PCDD/Fs in 1990 was estimated at 39 µg/t TEQ, based on data from SCU_RF plants in Croatia, Finland, Norway, Germany, and Sweden. The U.S. EF was significantly lower than the global average; according to the EPA, the EF remained constant at 3.7 µg/t TEQ in 1987, 1995, and 2000^14^. Entering the 21st century, based on PCDD/F emissions and SCU_RF production in Europe in 2005, the EF was approximately 20 µg/t TEQ^24-26^. However, a 2012 study on a Portuguese SCU_RF plant revealed an EF between 3.1 µg/t and 3.3 µg/t TEQ^27^. Research on PCDD/F emissions from SCU_RF in Asia began to emerge around 2010 and has gradually gained attention. In 2009, Ba et al. reported an EF of 15 µg/t TEQ for SCU_RF in China^28^. In 2012, based on emission estimates from two Chinese SCU_RF plants, the EFs were determined to be 1 µg/t TEQ and 9 µg/t TEQ, respectively^18^. By 2019, a study in China showed that an SCU_RF plant implementing pre-cleaning of raw materials had an emission factor as low as 0.3 µg/t TEQ^29^. The latest EF was found in the 2022 dioxin inventory published by the Ministry for the Environment in New Zealand^30^, which adopted an EF of PCDD/Fs at 3.5 µg/t TEQ to estimate PCDD/Fs emissions from SCU_RF.

According to the UNEP toolkit^13^, SCU_RF plants categorized as “with basic technology” (class 1), "well controlled" (class 2), and "optimized for PCDD/Fs control" (class 3) have PCDD/F EFs of 800 µg/t, 50 µg/t TEQ, and 5 µg/t TEQ, respectively. Based on the EFs from official documents and literature, the global EF levels for PCDD/Fs in SCU_RF processes generally fall at the class 3 level. Studies have also proven the similar formation pathways of PCDD/Fs, PCBs, and PCNs in thermal processes, including copper smelting processes^31^. Significant correlation is also found between EFs of PCDD/Fs and those of HCB and PeCBz^32^. Here, we adopt the EF of UPOPs for SCU_RF in class 3 for our preliminary estimation.

For PCDD/Fs, PCBs, and HCB, the EFs for SCU_RF in class 3 were adopted from the parameters provided in the toolkit. For PeCBz, we utilized an EF from a 2017 study that supplemented the toolkit's parameters^32^. For PCNs and HCBD, we collected EFs reported in existing studies on SCU_RF processes (Table S6). Ba et al. reported PCN EFs from five SCU_RF plants as 0.044, 0.008, 0.003, 0.097, and 1.989 µg TEQ/t^33^. A 2012 study identified PCN EFs of 0.035 and 0.264 µg TEQ/t from two SCU_RF plants^18^. These studies demonstrated a significant variation in the magnitude of PCN EFs for SCU_RF. Using clustering analysis, the EFs were grouped into three categories, and the average of the data in the lowest emission category was used as the EF for PCNs in class 3. The PCN EF for SCU_RF used in this study was determined to be 0.015 µg TEQ/t. For HCBD, one study^20^ has explored EFs of 10 SCU_RF facilities. Similarly, clustering analysis of HCBD EFs from ten SCU_RF plants showed three distinct distribution categories (Fig. S1). Following a similar approach to PCNs, the average EF of HCBD for SCU_RF in class 3 was determined at 12 µg/t for estimation in this study.

**Determination of EFs of UPOPs for SCU_DM**

No specific data are currently available for SCU_DM emissions of PeCBz, HCB, PCNs and HCBD, but data for PCDD/Fs and PCBs suggest SCU_DM EFs are about 2-3 times of PCU. EFs of UPOPs from SCU_DM are set twice of these of PCU where data are lacking.

**Text S4 Estimation of global and regional UPOP emissions**

The global and regional UPOP emissions from copper smelting under different scenarios from 2020 to 2050, were calculated using the following equations.

Global and reginal emissions of UPOPs *n* from copper smelting subsector *i* in year *m* is given by:

$E_{inm}=P_{im}*{Emission factor}_{in}$	(Equation 1)

$E_{inmf}=P_{imf}*{Emission factor}_{in}$	(Equation 2)

Where E_inm_ and E_inmf_ represent emissions of UPOPs *n* from copper smelting subsector *i* in year *m* globally and in region *f*, respectively. P_im_ and P_imf_ represent copper production from subsector *i* in year *m* for the world and for region *f*, respectively, and emission factor_in_ represents EF for UPOPs n in subsector i.

The total emission of UPOPs n from all copper smelting subsectors globally and in region *f* in year *m* (Etot_nm_ and Etot_nmf_) are calculated by:

${Etot}_{nm}=\sum_{i} E_{inm}$	(Equation 3)

${Etot}_{nmf}=\sum_{i} E_{inmf}$	(Equation 4)

**Fig. S1. Cluster analysis of SCU_RF EFs for HCBD and PCNs^16-20, 33^**

**Fig. S2. Reginal emissions of UPOPs under STEPS, APS and NEZ**

**Table S1. Copper production sourced from smelting subsectors from 2023 to 2050 in the STEPS, APS and NEZ (Mt)^4-6^**

| Scenarios | PCU (Mt) | | | | SCU_RF (Mt) | | | | SCU_DM (Mt) | | | |
| --- | --- | --- | --- | --- | --- | --- | --- | --- | --- | --- | --- | --- |
|  | 2023 | 2030 | 2040 | 2050 | 2023 | 2030 | 2040 | 2050 | 2023 | 2030 | 2040 | 2050 |
| STEPS | 22.4 | 21.9-24.8 | 21.3-24.6 | 21.4-24.2 | 4.4 | 5.3-8.9 | 8.5-11.9 | 12.3-14.9 | 6.2 | 9.0-9.2 | 11.8-11.9 | 15.1-15.2 |
| APS | 22.4 | 23.1-25.3 | 24.6-25.2 | 23.4-23.9 | 4.4 | 5.6-9.0 | 9.8-12.2 | 15.0-15.8 | 6.2 | 9.2-9.5 | 12.5-13.1 | 15.8-16.4 |
| NEZ | 22.4 | 26.7-27.1 | 27.9-28.8 | 23.0-28.8 | 4.4 | 5.6-9.1 | 10.1-14.9 | 15.6-19.4 | 6.2 | 9.7-10.5 | 13.5-15.4 | 16.3-19.8 |

**Table S2. Regional copper production from 2023-2050 under STEPS**

| Region | PCU | | | | SCU_RF | | | | SCU_DM | | | |
| --- | --- | --- | --- | --- | --- | --- | --- | --- | --- | --- | --- | --- |
|  | 2023 | 2030 | 2040 | 2050 | 2023 | 2030 | 2040 | 2050 | 2023 | 2030 | 2040 | 2050 |
| North America | 1.88 | 1.84–2.08 | 1.78–2.06 | 1.79–2.03 | 0.09 | 0.10–0.17 | 0.17–0.23 | 0.24–0.29 | 0.8 | 1.17–1.19 | 1.53–1.53 | 1.96–1.97 |
| Europe & Central Asia | 3.32 | 3.24–3.67 | 3.15–3.64 | 3.17–3.58 | 1.08 | 1.30–2.19 | 2.09–2.93 | 3.02–3.66 | 1.79 | 2.60–2.66 | 3.41–3.41 | 4.37–4.40 |
| Middle East & North Africa | 0.8 | 0.79–0.89 | 0.76–0.88 | 0.77–0.87 | 0.1 | 0.12–0.19 | 0.19–0.26 | 0.27–0.33 | 0.14 | 0.21–0.21 | 0.27–0.27 | 0.35–0.35 |
| Latin America & Caribbean | 3.04 | 2.97–3.37 | 2.89–3.34 | 2.90–3.28 | 0.04 | 0.05–0.09 | 0.09–0.12 | 0.12–0.15 | 1.09 | 1.59–1.62 | 2.08–2.08 | 2.66–2.68 |
| Sub-Saharan Africa | 0.07 | 0.07–0.08 | 0.07–0.08 | 0.07–0.08 | 0.01 | 0.02–0.03 | 0.03–0.04 | 0.04–0.05 | 0.03 | 0.05–0.05 | 0.07–0.07 | 0.08–0.09 |
| South Asia | 0.73 | 0.72–0.81 | 0.70–0.80 | 0.70–0.79 | 0.18 | 0.22–0.36 | 0.35–0.48 | 0.50–0.61 | 0.56 | 0.81–0.83 | 1.06–1.06 | 1.36–1.37 |
| East Asia & Pacific | 12.56 | 12.28–13.91 | 11.94–13.79 | 12.00–13.57 | 2.9 | 3.49–5.86 | 5.60–7.84 | 8.10–9.81 | 1.77 | 2.57–2.63 | 3.37–3.37 | 4.32–4.35 |

**Table S3. Regional copper production from 2023-2050 under APS**

| Region | PCU | | | | SCU_RF | | | | SCU_DM | | | |
| --- | --- | --- | --- | --- | --- | --- | --- | --- | --- | --- | --- | --- |
|  | 2023 | 2030 | 2040 | 2050 | 2023 | 2030 | 2040 | 2050 | 2023 | 2030 | 2040 | 2050 |
| North America | 1.88 | 1.94-2.12 | 2.06-2.11 | 1.96-2.00 | 0.09 | 0.11-0.18 | 0.19-0.24 | 0.29-0.31 | 0.8 | 1.19-1.23 | 1.62-1.70 | 2.05-2.13 |
| Europe & Central Asia | 3.32 | 3.42-3.75 | 3.64-3.73 | 3.47-3.54 | 1.08 | 1.38-2.21 | 2.41-3.00 | 3.69-3.88 | 1.79 | 2.66-2.75 | 3.62-3.79 | 4.57-4.75 |
| Middle East & North Africa | 0.8 | 0.83-0.91 | 0.88-0.90 | 0.84-0.86 | 0.1 | 0.12-0.20 | 0.21-0.27 | 0.33-0.35 | 0.14 | 0.21-0.22 | 0.29-0.30 | 0.36-0.38 |
| Latin America & Caribbean | 3.04 | 3.13-3.43 | 3.34-3.42 | 3.18-3.24 | 0.04 | 0.06-0.09 | 0.10-0.12 | 0.15-0.16 | 1.09 | 1.62-1.67 | 2.20-2.31 | 2.79-2.89 |
| Sub-Saharan Africa | 0.07 | 0.07-0.08 | 0.08-0.08 | 0.07-0.07 | 0.01 | 0.02-0.03 | 0.03-0.04 | 0.05-0.05 | 0.03 | 0.05-0.05 | 0.07-0.07 | 0.09-0.09 |
| South Asia | 0.73 | 0.76-0.83 | 0.80-0.82 | 0.77-0.78 | 0.18 | 0.23-0.37 | 0.40-0.50 | 0.61-0.64 | 0.56 | 0.83-0.86 | 1.13-1.18 | 1.42-1.48 |
| East Asia & Pacific | 12.56 | 12.95-14.19 | 13.79-14.13 | 13.12-13.40 | 2.9 | 3.69-5.93 | 6.46-8.04 | 9.88-10.41 | 1.77 | 2.63-2.72 | 3.57-3.75 | 4.50-4.69 |

**Table S4. Regional copper production from 2023-2050 under NEZ**

| Region | PCU | | | | SCU_RF | | | | SCU_DM | | | |
| --- | --- | --- | --- | --- | --- | --- | --- | --- | --- | --- | --- | --- |
|  | 2023 | 2030 | 2040 | 2050 | 2023 | 2030 | 2040 | 2050 | 2023 | 2030 | 2040 | 2050 |
| North America | 1.88 | 2.20-2.27 | 2.36-2.41 | 2.04-2.41 | 0.09 | 0.11-0.17 | 0.20-0.29 | 0.30-0.38 | 0.8 | 1.26-1.35 | 1.75-2.00 | 2.13-2.57 |
| Europe & Central Asia | 3.32 | 3.88-4.01 | 4.18-4.27 | 3.60-4.27 | 1.08 | 1.38-2.19 | 2.48-3.66 | 3.83-4.77 | 1.79 | 2.81-3.01 | 3.91-4.46 | 4.75-5.73 |
| Middle East & North Africa | 0.8 | 0.94-0.97 | 1.01-1.03 | 0.87-1.03 | 0.1 | 0.12-0.19 | 0.22-0.33 | 0.34-0.42 | 0.14 | 0.22-0.24 | 0.31-0.35 | 0.38-0.46 |
| Latin America & Caribbean | 3.04 | 3.56-3.68 | 3.83-3.91 | 3.30-3.91 | 0.04 | 0.06-0.09 | 0.10-0.15 | 0.16-0.20 | 1.09 | 1.71-1.83 | 2.38-2.72 | 2.89-3.49 |
| Sub-Saharan Africa | 0.07 | 0.08-0.08 | 0.09-0.09 | 0.08-0.09 | 0.01 | 0.02-0.03 | 0.03-0.05 | 0.05-0.06 | 0.03 | 0.05-0.06 | 0.08-0.09 | 0.09-0.11 |
| South Asia | 0.73 | 0.86-0.89 | 0.92-0.94 | 0.79-0.94 | 0.18 | 0.23-0.36 | 0.41-0.61 | 0.63-0.79 | 0.56 | 0.87-0.94 | 1.22-1.39 | 1.48-1.78 |
| East Asia & Pacific | 12.56 | 14.69-15.19 | 15.81-16.15 | 13.63-16.15 | 2.9 | 3.69-5.86 | 6.65-9.81 | 10.28-12.78 | 1.77 | 2.77-2.97 | 3.86-4.40 | 4.69-5.66 |

**Table S5. The regional share of copper production in each copper smelting sub-sector in 2021^12^**

| Region | PCU_share | SCU_RF_share | SCU_DM_share |
| --- | --- | --- | --- |
| North America | 8.38% | 1.95% | 12.98% |
| Europe & Central Asia | 14.81% | 24.58% | 28.94% |
| Middle East & North Africa | 3.59% | 2.19% | 2.30% |
| Latin America & Caribbean | 13.57% | 1.01% | 17.63% |
| Sub-Saharan Africa | 0.31% | 0.31% | 0.56% |
| South Asia | 3.27% | 4.07% | 9.01% |
| East Asia & Pacific | 56.07% | 65.87% | 28.59% |

**Table S6 EFs for the six UPOPS collected in previous research**

| UPOPs | Smelting Sub-sector | Factory | Emission factors (µg/t copper) | Emission factors (µg TEQ/t copper) | Reference |
| --- | --- | --- | --- | --- | --- |
| PCDD/Fs | PCU | PCU-1 | 0.4 | 0.04 | ^18^ |
|  |  | PCU-2 | 10 | 0.7 |  |
|  |  | PCU-3 | - | 0.004 | ^16^ |
|  |  | PCU-4 | - | 0.005 |  |
|  |  | PCU-5 | - | 0.01 | ^17^ |
|  |  | PCU-6 | - | 0.01 | ^19^ |
| PCBs | PCU | PCU-1 | - | 0.01 | ^17^ |
|  |  | PCU-2 | 5 | 0.002 | ^18^ |
|  |  | PCU-3 | 4.2 | 0.04 |  |
| PeCBz | PCU | PCU-1 | 53.1 | - | ^18^ |
|  |  | PCU-2 | 413 | - |  |
| HCB | PCU | PCU-1 | 37.7 | - | ^18^ |
|  |  | PCU-2 | 294 | - |  |
| HCBD | PCU | PCU-1 | n.d. | - | ^20^ |
|  |  | PCU-2 | 2.5 | - |  |
|  |  | PCU-3 | 0.5 | - |  |
|  |  | PCU-4 | 2.3 | - |  |
|  |  | PCU-5 | n.d. | - |  |
|  |  | PCU-6 | 2.2 | - |  |
|  | SCU_RF | SCu-1 | 9.8 | - | ^20^ |
|  |  | SCu-2 | 1.0 | - |  |
|  |  | SCu-3 | 8.8 | - |  |
|  |  | SCu-4 | 1.8 | - |  |
|  |  | SCu-5 | 4859.1 | - |  |
|  |  | SCu-6 | 1656.2 | - |  |
|  |  | SCu-7 | 35.4 | - |  |
|  |  | SCu-8 | 16.0 | - |  |
|  |  | SCu-9 | 17.3 | - |  |
|  |  | SCu-10 | 3.0 | - |  |
| PCNs | PCU | PCU-1 | 11.2 | 0.0002 | ^18^ |
|  |  | PCU-2 | 69 | 0.013 |  |
|  | SCU_RF | SCU-1 | 624 | 0.04 |  |
|  |  | SCU-2 | 246 | 0.008 |  |
|  |  | SCU-3 | 141 | 0.003 |  |
|  |  | SCU-4 | 7014 | 2.0 | ^33^ |
|  |  | SCU-5 | 1237 | 0.1 |  |
|  |  | SCU-6 | 244 | 0.04 | ^18^ |
|  |  | SCU-7 | 9154 | 0.3 |  |

**Table S7. EFs of copper smelting sub-sectors for each investigated UPOPs^16-20, 33^**

| UPOPs | PCU | SCU_DM | SCU_RF |
| --- | --- | --- | --- |
| PCDD/Fs^a^ | 0.010 (0.005-0.700) | 0.030 (0.015-2.100) | 5 (0.650-38.780) |
| PCBs^a^ | 0.018 (0.002-0.040) | 0.030 (0.003-0.067) | 0.3 (0.039-2.330) |
| PeCBz^a^ | 233 (53-413) | 466 (106-826) | 3000 (386-23292) |
| HCB^a^ | 165 (38-294) | 330 (76-588) | 1000 (129-7764) |
| PCNs^a^ | 0.007 (0.0002-0.013) | 0.013 (0.0004-0.026) | 0.015 (0.002-0.116) |
| HCBD^a^ | 2 (0.5-2.5) | 4 (1-5) | 12 (2-93) |

a: µg TEQ/t copper for PCDD/Fs, PCB and PCNs, µg/t copper for PeCBz, HCB and HCBD

References:

(1) Parpan, G.; Andrieu, B.; Vidal, O.; Delannoy, L.; Le Boulzec, H.; Gervais, M.; Jégourel, Y.; Delalande, S. Examining copper supply consistency in socioeconomic pathways: A mine-level dynamic approach. *Resources, Conservation and Recycling* **2026**, *225*,

(2) Klose, S.; Pauliuk, S. Sector-level estimates for global future copper demand and the potential for resource efficiency. *Resources, Conservation and Recycling* **2023**, *193*,

(3) Schipper, B. W.; Lin, H.-C.; Meloni, M. A.; Wansleeben, K.; Heijungs, R.; van der Voet, E. Estimating global copper demand until 2100 with regression and stock dynamics. *Resources, Conservation and Recycling* **2018**, *132*, 28-36

(4) Born, K.; Ciftci, M. M. The limitations of end-of-life copper recycling and its implications for the circular economy of metals. *Resources, Conservation and Recycling* **2024**, *200*,

(5) IEA. *Recycling of Critical Minerals*; 2024. <https://www.iea.org/reports/recycling-of-critical-minerals>.

(6) International Finance Corporation. *NET ZERO ROADMAP FOR COPPER AND NICKEL*; 2023. <https://ifcsia.org/>.

(7) Li, J.; Eheliyagoda, D.; Geng, Y.; Yang, Z.; Zeng, X. Examining the influence of copper recycling on prospective resource supply and carbon emission reduction. *Fundam Res* **2025**, *5* (2), 740-747

(8) Elshkaki, A.; Graedel, T. E.; Ciacci, L.; Reck, B. K. Copper demand, supply, and associated energy use to 2050. *Global Environmental Change* **2016**, *39*, 305-315

(9) Watari, T.; Nansai, K.; Nakajima, K. Major metals demand, supply, and environmental impacts to 2100: A critical review. *Resources, Conservation and Recycling* **2021**, *164*,

(10) International Copper Association. *Copper—The Pathway to Net Zero*; ICA, 2023. <https://internationalcopper.org/resource/copper-pathway-to-net-zero/>.

(11) Watari, T.; Northey, S.; Giurco, D.; Hata, S.; Yokoi, R.; Nansai, K.; Nakajima, K. Global copper cycles and greenhouse gas emissions in a 1.5 °C world. *Resources, Conservation and Recycling* **2022**, *179*,

(12) ICSG. *56th Meeting Environmental and Economic Committee*; 2024. <https://icsg.org/>.

(13) UNEP. *Toolkit for Identification and Quantification of Releases of Dioxins, Furans and Other Unintentional POPs*; 2013. <https://toolkit.pops.int/>.

(14) US EPA. *Update to An Inventory of Sources and Environmental Releases of Dioxin-Like Compounds in the United States for the Years 1987, 1995, and 2000* US EPA,,, 2013. <https://assessments.epa.gov/dioxin/document/&deid=235432>.

(15) Ministry of Environment Poland. *Inventory of Dioxin and Furan Release in Poland*; Ministry of Environment Poland,,, 2002. <https://bip.mos.gov.pl/>.

(16) Grochowalski, A.; Lassen, C.; Holtzer, M.; Sadowski, M.; Hudyma, T. Determination of PCDDs, PCDFs, PCBs and HCB emissions from the metallurgical sector in Poland. *Environ Sci Pollut Res Int* **2007**, *14* (5), 326-332

(17) Yu, B. W.; Jin, G. Z.; Moon, Y. H.; Kim, M. K.; Kyoung, J. D.; Chang, Y. S. Emission of PCDD/Fs and dioxin-like PCBs from metallurgy industries in S. Korea. *Chemosphere* **2006**, *62* (3), 494-501

(18) Nie, Z.; Liu, G.; Liu, W.; Zhang, B.; Zheng, M. Characterization and quantification of unintentional POP emissions from primary and secondary copper metallurgical processes in China. *Atmospheric Environment* **2012**, *57*, 109-115

(19) EEA. *EMEP/EEA air pollutant emission inventory guidebook 2023*; EEA, 2023. <https://efdb.apps.eea.europa.eu/?source=%7B%22query%22%3A%7B%22match_all%22%3A%7B%7D%7D%2C%22display_type%22%3A%22tabular%22%7D>.

(20) Zhao, C.; Yang, L.; Sun, Y.; Chen, C.; Huang, Z.; Yang, Q.; Yun, J.; Habib, A.; Liu, G.; Zheng, M.; Jiang, G. Atmospheric emissions of hexachlorobutadiene in fine particulate matter from industrial sources. *Nat Commun* **2024**, *15* (1), 4737

(21) Bond, T. C.; Streets, D. G.; Yarber, K. F.; Nelson, S. M.; Woo, J. H.; Klimont, Z. A technology‐based global inventory of black and organic carbon emissions from combustion. *Journal of Geophysical Research: Atmospheres* **2004**, *109* (D14),

(22) US EPA. *Guidance for Reporting the Dioxin and Dioxin-like Compounds Category*; US EPA,,, 2019. <https://www.epa.gov/toxics-release-inventory-tri-program/guidance-dioxin-and-dioxin-compounds-category>.

(23) UNEP. *National and Regional Emissions of PCDD/PCDF*; 1999. <https://www.unep.org/resources/report/dioxin-and-furan-inventories-national-and-regional-emissions-pcdd-pcdf>.

(24) Quass, U.; Fermann, M.; Broker, G. The European dioxin air emission inventory project--final results. *Chemosphere* **2004**, *54* (9), 1319-1327

(25) Commission, E. *EU Ferrous & Non-Ferrous Metals Industry*; European Commission, 2007. <https://eippcb.jrc.ec.europa.eu/reference/non-ferrous-metals-industries-0>.

(26) Soulier, M.; Glöser-Chahoud, S.; Goldmann, D.; Tercero Espinoza, L. A. Dynamic analysis of European copper flows. *Resources, Conservation and Recycling* **2018**, *129*, 143-152

(27) Antunes, P.; Viana, P.; Vinhas, T.; Rivera, J.; Gaspar, E. M. Emission profiles of polychlorinated dibenzodioxins, polychlorinated dibenzofurans (PCDD/Fs), dioxin-like PCBs and hexachlorobenzene (HCB) from secondary metallurgy industries in Portugal. *Chemosphere* **2012**, *88* (11), 1332-1339

(28) Ba, T.; Zheng, M.; Zhang, B.; Liu, W.; Xiao, K.; Zhang, L. Estimation and characterization of PCDD/Fs and dioxin-like PCBs from secondary copper and aluminum metallurgies in China. *Chemosphere* **2009**, *75* (9), 1173-1178

(29) Li, H.; Liu, W.; Tang, C.; Lei, R.; Zhu, W. Emission profiles and formation pathways of 2,3,7,8-substituted and non-2,3,7,8-substituted polychlorinated dibenzo-p-dioxins and dibenzofurans in secondary copper smelters. *Sci Total Environ* **2019**, *649*, 473-481

(30) Ministry for the Environment. *2022 Update of the New Zealand Inventory of Dioxin Emissions to Air, Land and Water, and Reservoir Sources*; Ministry for the Environment,,, 2022. <https://environment.govt.nz/publications/2022-update-of-the-new-zealand-inventory-of-dioxin-emissions-to-air-land-and-water-and-reservoir-sources/>.

(31) Jiang, X.; Liu, G.; Wang, M.; Zheng, M. Formation of Polychlorinated Biphenyls on Secondary Copper Production Fly Ash: Mechanistic Aspects and Correlation to Other Persistent Organic Pollutants. *Sci Rep* **2015**, *5*, 13903

(32) Gong, W.; Fiedler, H.; Liu, X.; Wang, B.; Yu, G. Emission factors of unintentional HCB and PeCBz and their correlation with PCDD/PCDF. *Environ Pollut* **2017**, *230*, 516-522

(33) Ba, T.; Zheng, M.; Zhang, B.; Liu, W.; Su, G.; Liu, G.; Xiao, K. Estimation and congener-specific characterization of polychlorinated naphthalene emissions from secondary nonferrous metallurgical facilities in china. *Environ. Sci. Technol.* **2011**, *44*, 2441–2446
